# Antithrombin, COVID-19, and Fresh Frozen Plasma Treatment

**DOI:** 10.4274/tjh.galenos.2021.2021.0155

**Published:** 2021-06-01

**Authors:** Rujitttika Mungmungpuntipantip, Viroj Wiwanitkit

**Affiliations:** 1 Private Academic Consultant, Bangkok, Thailand; 2 Honorary Professor, Dr DY Patil University, Pune, India

**Keywords:** Antithrombin, COVID-19, Fresh frozen plasma

## To the Editor,

We found the article entitled “Prognostic value of antithrombin levels in COVID-19 patients and impact of fresh frozen plasma treatment: a retrospective study” very interesting [1]. Considering antithrombin (AT) levels and fresh frozen plasma (FFP) in these patients, Anaklı et al. [1] concluded that “AT activity could be used as a prognostic marker for survival and organ failure in COVID-19-associated ARDS patients. AT supplementation therapy with FFP in patients with COVID-19-induced hypercoagulopathy may improve thrombosis prophylaxis and thus have an impact on survival” [1]. Indeed, plasma therapy is a widely discussed alternative option for management of severe coronavirus disease-19 (COVID-19). Some medical scientists have proposed the usefulness of non-convalescent plasma therapy. In a recent report, Bajpai et al. [2] found that the median improvement in PaO2/FiO2 in COVID-19 patients treated with non-convalescent plasma was significantly superior to results of FFP at 48 hours. Additionally, an important consideration for any kind of plasma therapy is the safety. The major consideration is possible pathogenic contamination in plasma [3]. Finally, the exact pathomechanism of COVID-19-related coagulopathy is not well clarified but it is believed to be an immunopathological process [4]. The use of FFP therapy is not direct management for the underlying immunological problem; a more appropriate management might be plasma exchange therapy [5].
