# Supplementary material for: Antithrombin, COVID-19, and Fresh Frozen Plasma Treatment
Source: Turk J Haematol. 2021 Jun 1;38(2):157–9. doi: 10.4274/tjh.galenos.2021.2021.0155 (PMC8171211; doi:10.4274/tjh.galenos.2021.2021.0155)
Supplement: Related texts

# REPLY FROM THE AUTHORS

We would like to thank the editor for the opportunity to respond to the issues raised in this letter and to clarify aspects of our methodology in relation to these concerns. In our study, we found that acute antithrombin (AT) deficiency may contribute to both the development of thrombosis and failure to achieve maintained therapeutic anticoagulation in patients with coronavirus disease-2019 (COVID-19). AT supplementation may increase the anticoagulant effect of heparin, so low AT activity levels (<75%) were treated with fresh frozen plasma (FFP) in COVID-19-related acute respiratory distress syndrome patients. We observed an increase in AT levels after FFP treatment (from 53% to 80%), and much higher levels were achieved in survivors than nonsurvivors (82% vs. 76%). Thromboembolic events (TEs) were not seen in those patients who had AT activity of ≥75% after FFP treatment and D-dimer levels were significantly reduced after supplementation [1]. In the present letter, the authors mention the usefulness of non-convalescent plasma therapy. However, there is no clear evidence about the usefulness of non-convalescent plasma therapy, the optimal dose, or the time point for this therapy [2]. Studies are limited to small case series, so it is not a drug that is used for a long time and the safety of plasma has not been confirmed. In past studies, there was no significant difference between convalescent plasma and FFP in terms of viral load, cytokine levels, mortality, duration of hospitalization or intensive care stay, and days free of mechanical ventilation [3]. The pathogenic contamination risk is also similar with FFP and finding a donor is not always possible.

A substantial proportion of COVID-19 patients develop thrombotic complications due to the uncontrolled immunothrombotic response. We aimed to prevent TEs and organ dysfunction related to hypercoagulability by using AT supplementation. However, AT concentrates are not available in Turkey, so we treated patients with FFP for AT supplementation. Therapeutic plasma exchange (TPE) has been used for rescue therapy in critically ill patients with COVID-19 who do not respond to conventional therapies [4]. Although used as alternative therapy in COVID-19 patients, extracorporeal therapies may lead to increased cytokine release, thrombosis, and disseminated intravascular coagulation. Studies about extracorporeal membrane oxygenation showed that continuous contact surface between the humoral and cellular components of the blood and the extracorporeal circuit may cause a systemic activation of coagulation and inflammation pathways [5]. Another problem is hemodynamic changes during TPE. Hypotension, arrhythmias, and shock can be seen during TPE. Hypersensitivity and pathogen contamination risk may be higher than in our protocol due to the need for higher FFP volumes for TPE. Also, the efficacy of drugs used for COVID-19 treatment may decrease during TPE because of the removal or dilution of the drugs by TPE. Blood supply difficulties and high costs are other problems related to TPE. It requires skilled staff, special equipment, and extra catheter insertion, so it may not be readily available in many locations. Furthermore, the time frame for TPE is controversial.

Globally, more than 100 million confirmed cases of COVID-19 have been recorded, with more than two million deaths [6]. Various therapeutic agents have been used for COVID-19 patients, all of which remain experimental. More studies need to be done for more effective treatment suggestions.

**İlkay Anaklı, Perihan Ergin Özcan, Özlem Polat, Günseli Orhun, Gülçin Hilal Alay, Verda Tuna, Emre Çeliksoy, Mehmet Kılıç, Mutlu Mercan, Achmet Ali, Sevgi Beşışık, Figen Esen**

## References

[1] Anaklı İ, Ergin Özcan P, Polat Ö, Orhun G, Alay GH, Tuna V, Çeliksoy E, Kılıç M, Mercan M, Ali A, Beşışık S, Esen F (2021). Prognostic value of antithrombin levels in COVID-19 patients and impact of fresh frozen plasma treatment: a retrospective study. Turk J Hematol.

[2] Balcells ME, Rojas L, Le Corre N, Martínez-Valdebenito C, Ceballos ME, Ferrés M, Chang M, Vizcaya C, Mondaca S, Huete Á, Castro R, Sarmiento M, Villarroel L, Pizarro A, Ross P, Santander J, Lara B, Ferrada M, Vargas-Salas S, Beltrán-Pavez C, Soto-Rifo R, Valiente-Echeverría F, Caglevic C, Mahave M, Selman C, Gazitúa R, Briones JL, Villarroel-Espindola F, Balmaceda C, Espinoza MA, Pereira J, Nervi B (2021). Early versus deferred anti-SARS-CoV-2 convalescent plasma in patients admitted for COVID-19: a randomized phase II clinical trial. PLoS Med.

[3] Bajpai M, Kumar S, Maheshwari A, Chhabra K, kale P, Gupta A, Narayanan A, Gupta E, Trehanpati N, Bihari C, Agarwal R, Gupta K, Gupta UK, Bhardwaj A, Kumar G, Islam M, Singh R, Yadav P, Maiwall R, Sarin SK. Efficacy of convalescent plasma therapy compared to fresh frozen plasma in severely ill COVID-19 patients: a pilot randomized controlled trial. medRxiv Preprints. Available at https://doi.org/10.1101/2020.10.25.20219337.

[4] Fernandez J, Gratacos-Ginès J, Olivas P, Costa M, Nieto S, Mateo D, Sánchez MB, Aguilar F, Bassegoda O, Ruiz P, Caballol B, Pocurull A, Llach J, Mustieles MJ, Cid J, Reverter E, Toapanta ND, Hernández-Tejero M, Martínez JA, Claria J, Fernández C, Mensa J, Arroyo V, Castro P, Lozano M;, Covid Clinic Critical Care (CCCC) Group (2020). Plasma exchange: an effective rescue therapy in critically ill patients with coronavirus disease 2019 infection. Crit Care Med.

[5] Von Bahr V, Millar JE, Malfertheiner MV, Ki KK, Passmore MR, Bartnikowski N, Redd MA, Cavaye M, Suen JY, McAuley DF, Fraser JF (2019). Mesenchymal stem cells may ameliorate inflammation in an ex vivo model of extracorporeal membrane oxygenation. Perfusion.

[6] World Health Organization. WHO Coronavirus Disease (COVID-19) Dashboard. Geneva, WHO, 2021. Available at.
